# Public Views About Involvement in Decision-Making on Health Data Sharing, Access, Use and Reuse: The Importance of Trust in Science and Other Institutions

**DOI:** 10.3389/fpubh.2022.852971

**Published:** 2022-05-10

**Authors:** Ngozi Nwebonyi, Susana Silva, Cláudia de Freitas

**Affiliations:** ^1^Laboratório para a Investigação Integrativa e Translacional em Saúde Populacional (ITR), Porto, Portugal; ^2^Departamento de Ciências da Saúde Pública e Forenses e Educação Médica, Faculdade de Medicina da Universidade do Porto (FMUP), Porto, Portugal; ^3^Departamento de Sociologia, Instituto de Ciências Sociais, Universidade do Minho, Braga, Portugal; ^4^Centro em Rede de Investigação em Antropologia, Universidade do Minho, Braga, Portugal; ^5^EPIUnit - Instituto de Saúde Pública, Universidade do Porto, Porto, Portugal; ^6^Centre for Research and Studies in Sociology, University Institute of Lisbon (ISCTE-IUL), Lisbon, Portugal

**Keywords:** public involvement, data governance, trust, research trustworthiness, data sharing, data access, data reuse, rare diseases

## Abstract

**Background:**

Data-intensive and needs-driven research can deliver substantial health benefits. However, concerns with privacy loss, undisclosed surveillance, and discrimination are on the rise due to mounting data breaches. This can undermine the trustworthiness of data processing institutions and reduce people's willingness to share their data. Involving the public in health data governance can help to address this problem by imbuing data processing frameworks with societal values. This study assesses public views about involvement in individual-level decisions concerned with health data and their association with trust in science and other institutions.

**Methods:**

Cross-sectional study with 162 patients and 489 informal carers followed at two reference centers for rare diseases in an academic hospital in Portugal (June 2019–March 2020). Participants rated the importance of involvement in decision-making concerning health data sharing, access, use, and reuse from “not important” to “very important”. Its association with sociodemographic characteristics, interpersonal trust, trust in national and international institutions, and the importance of trust in research teams and host institutions was tested.

**Results:**

Most participants perceived involvement in decision-making about data sharing (85.1%), access (87.1%), use (85%) and reuse (79.9%) to be important or very important. Participants who ascribed a high degree of importance to trust in research host institutions were significantly more likely to value involvement in such decisions. A similar position was expressed by participants who valued trust in research teams for data sharing, access, and use. Participants with low levels of trust in national and international institutions and with lower levels of education attributed less importance to being involved in decisions about data use.

**Conclusion:**

The high value attributed by participants to involvement in individual-level data governance stresses the need to broaden opportunities for public participation in health data decision-making, namely by introducing a meta consent approach. The important role played by trust in science and in other institutions in shaping participants' views about involvement highlights the relevance of pairing such a meta consent approach with the provision of transparent information about the implications of data sharing, the resources needed to make informed choices and the development of harm mitigation tools and redress.

## Introduction

Health care quality improvement can be bolstered by data-intensive and needs-driven research (1). The use of big health data promises to transform biomedical and health care research and to deliver substantial public health benefits that range from disease risk prediction and prevention to the discovery of new therapies for untreatable health conditions, as are many rare diseases (2, 3). However, mounting reports of data breaches and mismanagement have generated concern for privacy loss, undisclosed surveillance, and discrimination (4–6). These concerns can undermine public trust in data processing organizations (e.g., governmental, care and research institutions), which is key in shaping public attitudes toward data sharing and use (7, 8). For instance, large-scale health data projects such as care.data and the Clinical Practice Research Datalink (CPRD) in the UK have failed to accomplish their goals because they could not achieve public trust and acceptability, despite promising benefits to health care and the public. Studies of the public's opinion suggest that these projects failed due to public concerns about informed consent, limited trust in data security and privacy, lack of communication on how data linkage would work, and the undisclosed involvement of commercial and private companies (7, 9, 10). Concerns such as these can evolve into a more generalized perceived lack of institutional trustworthiness, which can limit people's willingness to share their data for research and to concede to its (re)use (11–13). There is, therefore, an imminent need for optimizing governance strategies to promote safe, acceptable, and beneficial uses of data in health research.

International policy agencies recommend the involvement of the public to ensure that data processing frameworks are consistent with societal values and individuals' expectations for the protection and use of their data (14, 15). Public involvement is also substantiated by ethical arguments that center on the fair distribution of the benefits arising from data use (16, 17). Furthermore, it has been argued that public involvement exercises can help foster authentic dialogue between researchers and publics, enhance accountability among data stewards on the governance chain and increase research trustworthiness, all of which are vital for ensuring and sustaining public trust in science (7).

Public involvement in health data governance entails awareness raising, consultation, partnering with and/or empowering of members of the public to participate in research and governance practices and it can be set in motion through a variety of methods including deliberative polls, citizen juries, participatory appraisals, scenario-based workshops, and focus groups (18). Data holders can also participate via participant-led data cooperatives (e.g., Open Humans, PEER Network, MIDATA) that enable them to share and aggregate their data while keeping control over its uses (19–23).

At the individual level, public involvement can be fostered by enabling lay people to participate in decisions about particular aspects of data governance, including whether they want to share their data (*data sharing*), with whom they want to share their data with (*data access*), for what purposes it can be used (*data use*) and whether data can be shared for purposes other than those for which they were originally collected (*data reuse*). These individual-level decisions are typically enacted through different types of informed consent procedures (24, 25).

Broad consent offers data donors limited opportunities for decision-making beyond the initial decision of sharing data. In this type of consent potential data donors are asked consent to sharing data for purposes that may not yet be entirely specified but whose core aim is known to the public. As such, it differs from blanket consent in which shared data can be used without any restrictions (26, 27). Specific consent enables data donors to have more control over their data by enabling them to decide who uses the data and for what purposes, within the scope of a specific project or a set of similar research initiatives. Both broad and specific consent are requested at the moment people are asked to share data, usually at one single time-point. Dynamic consent, on the other hand, enables a higher degree of involvement in decision-making by allowing data donors to define and modify consent preferences over time, including decisions about the possible reuse of their data (28, 29). The latter implies the creation of interactive platforms that enable data subjects to be notified of requests to use their data and to be re-contacted to proceed with making a decision regarding consent (30, 31). Ploug and Holm (32) argue that adopting a dynamic consent approach can lead to the routinization of consent and even to “consent fatigue”, as participants will likely receive large amounts of consent requests each requiring analysis of an entire project. Alternatively, they propose a meta consent model, which combines the broad and dynamic consent models with additional options for blanket consent and blanket refusal. In this type of model, data donors choose what type of consent (e.g., broad, blanket, dynamic, refusal) they would like to provide for the reuse of their data in future projects. Such a choice can be done both according to data type (e.g., electronic patient records, tissue, health data, non-health data) and to the context of data use (e.g., public vs. private, commercial vs. non-commercial, national vs. international). For instance, if data donors choose dynamic consent for health-data reuse by the commercial sector, they will be asked for specific consent each time data for a new project is requested in that domain. If instead they choose broad consent for electronic patient records reuse by public health institutions, they will be asked for consent only when a new project falls outside the scope of projects they already gave consent to (32).

Opportunities for public involvement have expanded substantially in the past decade and there is a growing interest in understanding whether patients, and other members of the public, value involvement in individual-level decisions about health data sharing, access, use and reuse (33–40). Ludman et al. (38) found that research participants wanted to decide whether their previously shared data could be submitted to a new database through active engagement in reconsenting procedures despite “their extraordinary trust in the research team” (32). Similarly, another study showed that patients would like to be re-contacted to decide on the reuse of their data and that not being given the opportunity to reconsent would be perceived as a threat to individual and group autonomy (33). Courbier et al. found that patients and their family members would like to keep control over their shared data and that about half would not delegate the decision about whom their data will be shared with to an ethics committee (34). And a study involving research participants in four European countries showed they were supportive of de-identified data reuse if they were involved in decision-making about data sharing and access, namely by retaining control to withdraw their data at any time (35).

Most existing studies address the multiple aspects of individual-level data governance independently and few have explored how trust in research initiatives influences the value bestowed by different publics on involvement in data decision-making (34, 38). In this study, we assess the views of rare disease patients and their informal carers about being involved in decisions regarding data sharing, access, use and reuse with a focus on the role played by trust in science and other institutions. Most rare diseases have no treatment and specific rare disease populations are very small and scattered geographically (34, 41). Data sharing within and between countries is therefore essential for enabling research that can advance the development of accurate diagnoses and therapies (42). However, this type of research requires a combination of genetic and phenotypic information which presents a high privacy risk for these patients and their relatives. Assessing rare disease patients and carers' views about involvement in decision-making concerned with their data can help in designing a data governance structure suitable to meet their needs and expectations from biomedical and health care research and to enhance the trustworthiness of institutions involved in research (16, 43).

## Materials and Methods

### Participants

This observational and cross-sectional study is part of a mixed methods project focusing on public involvement in health data governance whose protocol is described elsewhere (44). For the purposes of this paper, participants include people with rare diseases and their informal carers who are both stakeholders directly involved in decisions regarding their own data sharing, access, use and reuse for biomedical and health care research. Participants were consecutively recruited from two Reference Centers for Rare Diseases at the University Hospital Center S. João (UHCSJ), in Portugal, between June 2019 and March 2020. Following a consultation, patients aged 12 years and above and their carers were handed a study information leaflet by a health professional. Subsequently, they were invited to participate in the study by a researcher who clarified any arising doubts or questions. Those who decided to participate were accompanied to a private setting, where they read and signed the informed consent. Underage participants who agreed to participate gave verbal consent and the informed consent form was signed by their legal representatives. All participants were asked to fill in a self-administered questionnaire individually.

Of the 728 people invited, 77 refused to take part in the study due to unwillingness to participate (*n* = 37), lack of time (*n* = 34), lack of consent from the legal tutor (*n* = 3), limited literacy (*n* = 2) and emotional distress following diagnosis (*n* = 1). In total, 651 people (162 patients and 489 carers) agreed to participate (response rate: 89.4%).

### Data Collection

The structured questionnaire was developed by the research team based on a review of literature and existing instruments related to the research topic. The questionnaire was pretested by specialists with combined experience as professionals, informal carers and researchers (social and health sciences) and subsequently piloted by a group of patients and carers. The full questionnaire is available online [see (44)].

The assessment of the importance attributed by participants to involvement in decisions about their own health data sharing, access, use and reuse was based on the analysis of answers to four questions: 1) how important is it that you decide whether your data is shared for research purposes (*data sharing*); 2) how important is it that you decide whom your data is shared with (*data access*); 3) how important is it that you decide for what purposes your data is used for (*data use*); and, 4) how important is it that you decide whether your data can be used for purposes other than those for which it was initially collected (*data reuse*). The level of importance was rated using a 5-point Likert scale, ranging from “very important” to “not important” (range 1–5). For this analysis, the variables were categorized into “important” (including participants who answered “important” and “very important”) and “other” (including “not important”, “slightly important” and “moderately important”). This study included 637 participants (159 patients and 478 carers), with available data on all the above-mentioned outcomes.

Data on sociodemographic characteristics (sex, age, educational level, marital status, occupation, and perceived income adequacy), as well as participants' involvement with patient organizations were collected. Occupations were classified according to the Portuguese Classification of Occupations 2011 (45) and grouped into four categories: (1) upper-white-collar, including executive civil servants, industrial directors and executives, professionals and scientists, middle management and technicians; (2) lower-white-collar, including administrative and related workers, service and sales workers; (3) blue-collar, which includes farmers and skilled agricultural workers, fisheries workers, skilled workers, craftsmen and similar, machine operators and assembly workers, and unskilled workers; and (4) other, including students, unemployed, domestic workers, participants on disability pension or on paid/unpaid leave, retired and informal carers or members of a foster family. Perceived income adequacy was measured through the question “When thinking of your household income, would you say that your household is able to make ends meet?”. Participants could check one of the following answer categories: insufficient, caution with expenses, enough to make ends meet, and comfortable.

Interpersonal trust, trust in national institutions and trust in international institutions were measured through ten self-administered questions based on the European Social Survey (ESS) rated on a scale from 0 to 10. Interpersonal trust was measured by three questions: “Generally speaking, would you say that most people can be trusted or that you can't be too careful in dealing with people?”; “Do you think that most people would try to take advantage of you if they got the chance, or would they try to be fair?”; and “Would you say that most of the time people try to be helpful or that they are mostly looking out for themselves?”. As reported in another study (46) principal component analysis to these three questions produced a single component, explaining 70% of the variance. Institutional trust was measured by asking participants how they trusted national institutions such as a country's parliament, the legal system, the police, politicians, and political parties, as well as international institutions, namely the European Parliament and the United Nations. Principal component analysis of the dataset shows that the variables are well suited for constructing two indexes, one for trust in national institutions and another for trust in international institutions. The total score of the rating scales is divided by the number of valid responses to make the indexes ranging from 0 to 10, with higher scores indicating higher levels of trust.

The views of patients and carers about the importance of trust in research host institutions and in research teams in decisions regarding data sharing were assessed using a 5-point Likert scale ranging from “not important” to “very important” (range: 0–4) for the question: “There are some aspects people consider important to decide if they will share their health data for scientific research. If you had to make such decision, how important would you rate the following aspects: (1) trust in the institution hosting the research; (2) trust in the team conducting the research”. For this analysis, the answers were dichotomized as “very important” and “other” (all other answers).

### Data Analysis

Categorical variables are presented as counts and proportions, while continuous variables were summarized as medians and interquartile range (P25–P75). The Chi-square test or the Fisher exact test, as well as the Mann-Whitney test were used, as appropriate, to assess the associations and mean differences between the explanatory variables and the outcomes. Statistical significance was set at a value of *p* < 0.01. The statistical analyses were performed using the software IBM SPSS Statistics for Windows, version 27.0 (IBM Corp., Armonk, N.Y., USA).

## Results

The characteristics of the participants and their views about involvement in decision-making on health data sharing, access, use, and reuse are presented in Tables 1 and 2, respectively. Most participants attained 12 or less years of education (75.6%) and were not involved with patient's organizations (94.9%). Almost 80% of the carers were female, while over 53% of the patients were male. Carers were older (>30 years) than patients (87.2 vs. 15.1%) and more frequently married or living with a partner (78.0 vs. 9.5%). More than half of carers perceived their income as insufficient (56.2%), while 64.6% of patients considered it comfortable/enough to make ends meet. About three quarters of the carers and two thirds of the patients perceived trust in research host institutions and trust in research teams as very important issues when making decisions about sharing data. Participants presented low levels of trust in national institutions (Median [P25–P75] 3.5 [1.8–5.2]), increasing slightly for trust in international institutions (Median [P25–P75] 5.0 [2.5–7.0]) and interpersonal trust (Median [P25–P75] 4.7 [3.0–6.7]).

**Table 1:** Characterization of the participants, stratified by people with rare diseases and their informal carers.

**Participants**	**Total (*N =* 637)**	**Patients (*n =* 159)**	**Carers (*n =* 478)**
Sex, *n* (%)			
Female	453 (71.1)	75 (47.2)	378 (79.1)
Male	184 (28.9)	84 (52.8)	100 (20.9)
Age (years), *n* (%)			
<18	92 (14.6)	92 (57.9)	-
18–30	103 (16.4)	43 (27.0)	60 (12.8)
>30	434 (69.0)	24 (15.1)	410 (87.2)
Educational level (years), *n* (%)			
≤ 12	476 (75.6)	151 (95.6)	325 (68.9)
>12	154 (24.4)	7 (4.4)	147 (31.1)
Marital status, *n* (%)			
Married/living with partner	384 (60.9)	15 (9.5)	369 (78.0)
Other	247 (39.1)	143 (90.5)	104 (22.0)
Occupation, *n* (%)			
Upper white-collar	148 (24.6)	5 (3.2)	143 (32.3)
Lower white-collar	116 (19.3)	8 (5.1)	108 (24.4)
Blue-collar	87 (14.5)	9 (5.7)	78 (17.6)
Other	250 (41.6)	136 (86.1)	114 (25.7)
Perceived income adequacy, *n* (%)			
Insufficient/Caution with expenses	315 (51.3)	51 (35.4)	264 (56.2)
Enough to make ends meet/comfortable	299 (48.7)	93 (64.6)	206 (43.8)
Involvement in patient organizations, *n* (%)			
No	598 (94.9)	154 (98.1)	444 (93.9)
Yes	32 (5.1)	3 (1.9)	29 (6.1)
Trust in research host institution, *n* (%)			
Very important	461 (73.6)	107 (67.7)	354 (75.6)
Other	165 (26.4)	51 (32.3)	114 (24.4)
Trust in research team, *n* (%)			
Very important	448 (71.5)	103 (65.6)	345 (73.4)
Other	179 (28.5)	54 (34.4)	125 (26.6)
Trust in national institutions, Md (P25-P75)	3.5 (1.8–5.2)	4.5 (2.0–6.0)	3.4 (1.8–5.0)
Trust in international institutions, Md (P25-P75)	5.0 (2.5–7.0)	6.5 (3.0–8.0)	5.0 (2.0–7.0)
Interpersonal trust, Md (P25-P75)	4.7 (3.0–6.7)	4.7 (2.4–6.7)	4.7 (3.0–6.4)

*In each variable, the total may not add 637 participants, 159 patients or 478 carers due to missing values. The proportions may not add 100 due to rounding*.

**Table 2:** Participants' views about involvement in decision-making regarding health data sharing, access, use and reuse.

**Involvement in decision-making regarding**	**Total** **(*N =* 637)** ***n* (%)**	**Patients** **(*n =* 159)** ***n* (%)**	**Carers** **(*n =* 478)** ***n* (%)**
Data sharing			
Not important	14 (2.2)	6 (3.8)	8 (1.7)
Slightly important	14 (2.2)	6 (3.8)	8 (1.7)
Moderately important	67 (10.5)	28 (17.6)	39 (8.2)
Important	286 (44.9)	62 (39.0)	224 (46.9)
Very important	256 (40.2)	57 (35.8)	199 (41.6)
Data access			
Not important	13 (2.0)	7 (4.4)	6 (1.3)
Slightly important	15 (2.4)	7 (4.4)	8 (1.7)
Moderately important	54 (8.5)	26 (16.4)	28 (5.9)
Important	265 (41.6)	55 (34.6)	210 (43.9)
Very important	290 (45.5)	64 (40.3)	226 (47.3)
Data use			
Not important	7 (1.1)	2 (1.3)	5 (1.0)
Slightly important	17 (2.7)	7 (4.4)	10 (2.1)
Moderately important	71 (11.1)	26 (16.4)	45 (9.4)
Important	271 (42.5)	62 (39.0)	209 (43.7)
Very important	271 (42.5)	62 (39.0)	209 (43.7)
Data reuse			
Not important	13 (2.0)	6 (3.8)	7 (1.5)
Slightly important	19 (3.0)	6 (3.8)	13 (2.7)
Moderately important	96 (15.1)	31 (19.5)	65 (13.6)
Important	255 (40.0)	67 (42.1)	188 (39.3)
Very important	254 (39.9)	49 (30.8)	205 (42.9)

*The proportions may not add 100 due to rounding*.

Most participants considered it important or very important to be involved in decisions concerned with health data sharing (85.1%), access (87.1%), use (85%) and reuse (79.9%). This trend was observed among both patients and carers (Table 2).

Carers and older participants stated more frequently the importance of being involved in decision-making regarding data sharing and data access (Table 3). More educated participants revealed a statistically significant tendency to attribute more importance to participation in decisions about data use, while participants with the lowest levels of trust in national and international institutions (Median [P25–P75]: 2.2[0.8–4.0]) and 3.0[1.0–5.0], respectively) were less likely to value such type of involvement. Participants who considered trust in research host institutions as very important rated higher the importance of being involved in decisions about data sharing, data access, data use, and data reuse. A similar position was primarily expressed by participants who valued trust in research teams for data sharing, data access, and data use.

**Table 3:** Factors influencing participants' views about involvement in decision-making regarding health data sharing, access, use and reuse.

	**Total**	**Data sharing**		**Data access**		**Data use**		**Data reuse**	
		**Important^**a**^** ***n* (%)**	**Other^**b**^ *n* (%)**	**Important^**a**^** ***n* (%)**	**Other^**b**^ *n* (%)**	**Important^**a**^** ***n* (%)**	**Other^**b**^ *n* (%)**	**Important^**a**^** ***n* (%)**	**Other^**b**^ *n* (%)**
	637	542 (85.1)	95 (14.9)	555 (87.1)	82 (12.9)	542 (85.1)	95 (14.9)	509 (79.9)	128 (20.1)
Type of participant									
Patient	159	119 (74.8)^*^	40 (25.2)^*^	119 (74.8)^*^	40 (25.2)^*^	124 (78.0)	35 (22.0)	116 (73.0)	43 (27.0)
Carer	478	423 (88.5)^*^	55 (11.5)^*^	436 (91.2)^*^	42 (8.8)^*^	418 (87.4)	60 (2.6)	393 (82.2)	85 (17.8)
Sex									
Female	453	393 (86.8)	60 (13.2)	405 (89.4)	48 (10.6)	394 (87.0)	59 (13.0)	371 (81.9)	82 (18.1)
Male	184	149 (81.0)	35 (19.0)	150 (81.5)	34 (18.5)	148 (80.4)	36 (19.6)	138 (75.0)	46 (25.0)
Age (years)									
<18	92	67 (72.8)^*^	25 (27.2)^*^	66 (71.7)^*^	26 (28.3)^*^	69 (75.0)	23 (25.0)	69 (75.0)	23 (25.0)
18-30	103	86 (83.5)^*^	17 (16.5)^*^	93 (90.3)^*^	10 (9.7)^*^	88 (85.4)	15 (14.6)	85 (82.5)	18 (17.5)
>30	434	384 (88.5)^*^	50 (11.5)^*^	389 (89.6)^*^	45 (10.4)^*^	379 (87.3)	55 (12.7)	350 (80.6)	84 (19.4)
Educational level (years)									
≤ 12	476	397 (83.4)	79 (16.6)	404 (84.9)	72 (15.1)	389 (81.7)^*^	87 (18.3)^*^	372 (78.2)	104 (21.8)
>12	154	141 (91.6)	13 (8.4)	145 (94.2)	9 (5.8)	148 (96.1)^*^	6 (3.9)^*^	133 (86.4)	21 (13.6)
Marital status									
Married/living with partner	384	342 (89.1)	42 (10.9)	348 (90.6)	36 (9.4)	338 (88.0)	46 (12.0)	318 (82.8)	66 (17.2)
Others	247	197 (79.8)	50 (20.2)	202 (81.8)	45 (18.2)	200 (81.0)	47 (19.0)	188 (76.1)	59 (23.9)
Occupation									
Upper white-collar	148	137 (92.6)	11 (7.4)	137 (92.6)	11 (7.4)	138 (93.2)	10 (6.8)	119 (80.4)	29 (19.6)
Lower white-collar	116	99 (85.3)	17 (14.7)	102 (87.9)	14 (12.1)	98 (84.5)	18 (15.5)	91 (78.4)	25 (21.6)
Blue-collar	87	69 (79.3)	18 (20.7)	76 (87.4)	11 (12.6)	70 (80.5)	17 (19.5)	68 (78.2)	19 (21.8)
Other	250	204 (81.6)	46 (18.4)	205 (82.0)	45 (18.0)	205 (82.0)	45 (18.0)	199 (79.6)	51 (20.4)
Perceived income adequacy									
Insufficient/Caution with expenses	315	265 (84.1)	50 (15.9)	281 (89.2)	34 (10.8)	265 (84.1)	50 (15.9)	253 (80.3)	62 (19.7)
Enough to make ends meet/comfortable	299	259 (86.6)	40 (13.4)	253 (84.6)	46 (15.4)	258 (86.3)	41 (13.7)	238 (79.6)	61 (20.4)
Involvement with patients' organizations									
No	598	508 (84.9)	90 (15.1)	522 (87.3)	76 (12.7)	508 (84.9)	90 (15.1)	477 (79.8)	121 (20.2)
Yes	32	31 (96.9)	1 (3.1)	29 (90.6)	3 (9.4)	31 (96.9)	1 (3.1)	29 (90.6)	3 (9.4)
Trust in research host institution									
Very important	461	412 (89.4)^*^	49 (10.6)^*^	423 (91.8)^*^	38 (8.2)^*^	421 (91.3)^*^	40 (8.7)^*^	385 (83.5)^*^	76 (16.5)^*^
Other	165	121 (73.3)^*^	44 (26.7)^*^	121 (73.3)^*^	44 (26.7)^*^	111 (67.3)^*^	54 (32.7)^*^	115 (69.7)^*^	50 (30.3)^*^
Trust in research team									
Very important	448	401 (89.5)^*^	47 (10.5)^*^	410 (91.5)^*^	38 (8.5)^*^	405 (90.4)^*^	43 (9.6)^*^	369 (82.4)	79 (17.6)
Other	179	132 (73.7)^*^	47 (26.3)^*^	135 (75.4)^*^	44 (24.6)^*^	128 (71.5)^*^	51 (28.5)^*^	131 (73.2)	48 (26.8)
Trust in national institutions, Md (P25-P75)	3.5 (1.8–5.2)	3.8 (1.8–5.4)	2.4 (1.0–3.8)	3.8 (1.8–5.3)	2.6 (1.3–5.0)	3.8 (2.0–5.4)^*^	2.2 (0.8–4.0)^*^	3.6 (1.8–5.2)	3.2 (1.6–5.3)
Trust in international institutions, Md (P25–P75)	5.0 (2.5–7.0)	5.0 (2.5–7.0)	3.5 (1.0–5.5)	5.0 (2.5–7.0)	3.0 (1.0–7.0)	5.0 (2.5–7.0)^*^	3.0 (1.0–5.0)^*^	5.0 (2.5–7.0)	4.5 (2.0–7.0)
Interpersonal trust, Md (P25–P75)	4.7 (3.0–6.7)	4.7 (3.0–6.7)	3.7 (2.0–6.0)	4.7 (3.0–6.3)	5.0 (2.3–6.7)	4.7 (3.0–6.3)	3.7 (2.0–6.7)	4.7 (3.0–6.3)	4.8 (3.0-6.7)

a *Includes participants who answered “important” and “very important”*;

b *includes participants who answered, “not important”, “slightly important” and “moderately important”*;

* *p < 0.001. In each variable, the total may not add 637 participants, 159 patients or 478 carers due to missing values. The proportions may not add 100 due to rounding*.

## Discussion

The majority of people affected by rare diseases who were surveyed placed a high value on opportunities for involvement in decisions about health data sharing, access, use and reuse (ranging between 80–87%). These views differ from those of other publics such as people with diabetes among whom less than 50% considered important to decide what type of data can be shared and with whom (33). However, they are echoed by rare diseases communities across Europe who expressed a strong desire in keeping control over their shared data throughout the data processing cycle (80%) (34). Difficulties in obtaining diagnoses, the absence of cures, and oftentimes of treatment, inspire a firm commitment on the part of rare disease patients and their carers toward advancing research, which is further strengthened by a perceived need to optimize the use of scarce biospecimens and research resources (34, 36, 40, 42, 47). These challenges may explain rare diseases participants' eagerness to engage in decisions about how their data should be governed. Playing an active role in deciding what data can be shared, with whom and for which purposes can help to not only reorient governance frameworks to become more commensurate with their values and preferences, but also directly impact their lives, and those of future generations, by driving research and care to meet their specific needs (34, 40).

Our findings also show a strong positive association between the value attributed to trust in science and the value attributed to public involvement in data governance. Participants who ascribed a high degree of importance to trust in research institutions when choosing whether to share their data were significantly more likely to value involvement across the full spectrum of aspects related with individual-level data governance (data sharing, access, use and reuse). A similar pattern was found for trust in researchers and involvement in decisions about data sharing, access and use. These findings resonate with Aitken et al. (7) argument that public involvement is one out a set of institutional arrangements that are central in ensuring the trustworthiness of research, which, in turn, is required to foster public trust in science. Scientific initiatives guided by participatory ideals privilege reciprocity and acknowledge participants' expectations, needs and agency, not least by facilitating a people-centered approach to consent that enables data donors to choose from blanket, broad or dynamic consent models (i.e. meta consent) (32). Dynamic consent approaches afford participants an ongoing opportunity to decide the conditions in which their data can be shared, accessed, used and reused, over time and across a range of research initiatives and settings. These approaches also contribute to the establishment of ongoing communication with, and feedback from, researchers that can give rise to more substantive participatory initiatives (e.g., public deliberation exercises; public engagement in data access committees) (29, 48–51). Such participatory initiatives carry potential to increase research transparency and to promote accountability by enabling researchers and diverse publics to come together and build dialogic relationships that are essential for uncovering existing concerns and imbuing systems of data governance with public values and the mechanisms needed to ensure checks and balances, oversight and redress for misconduct (7, 52, 53). However, while public involvement can enhance research trustworthiness, (7, 54, 55), a minimum level of public trust in science has to be present for public involvement to unfold (49). Our study corroborates these findings by showing that rare diseases patients and their carers are significantly more likely to value involvement in health data governance when they hold trust in science in high regard.

Following a wider international trend (56–58), the Portuguese population has reported relatively high levels of trust in science (56). Yet, its level of trust in other institutions, including the European Parliament, national government, and the legal and health care systems, tends to be substantially lower (59–61). Trust in national institutions is related to citizens' perception of how effective institutions are in attending to their needs. For example, in Portugal, citizens who perceive government to be less effective and trustworthy are also less satisfied with the health system (59). Participants in our study also expressed low trust in national institutions and, to a lesser extent, in international institutions. Importantly, our study further shows that participants with the lowest levels of trust in national and international institutions attributed significantly less importance to getting involved in decision-making about how their data can be used. This trend may find explanation in the idea that public involvement is unlikely to inspire reciprocal partnerships and lead to transformative change in institutions perceived to be opaque, irresponsive, and unaccountable (62). Effecting change that is transformative requires the development of trusting relationships between institutional stakeholders and lay members of the public, the ability to accommodate and build on different types of knowledge and expertise and a thorough commitment to attending to the needs, and responding to the concerns, of the various parties involved (63). Where institutions fail to cultivate trust, incentives for involvement may wane or disappear altogether (49). Participatory exercises demand time, skills, and the confidence that the efforts made are grounded on transparent information and can foster the change needed to engender meaningful partnerships and ensure accountability (7). When these conditions are not met the drive for participation tends to plummet.

Carers and older participants in our study were more prone to value involvement in decisions about data sharing and data access. These findings align with those of an international survey carried out with people affected by rare diseases that found that participants identifying as patient representatives and older respondents were both more likely to perceive health-related information as sensitive and to want to retain control over who accesses their information, how and why (34).

Finally, our study shows that participants with lower levels of education attributed significantly less importance to involvement in decision-making about the purposes for which their data can be used. This finding may be pointing to an unequal distribution of the resources needed to make informed decisions about data use (e.g., health and digital literacy, access to digital devices, communication and negotiation skills). Big data, machine learning and artificial intelligence have contributed to expand the purposes of biomedical and healthcare research to a multitude of fast-evolving fields (64). Increasingly, research endeavors focus on issues that lay people may not be familiar with and feel wary to express opinions about (e.g., gene therapy) (65). Disregard for the needs of publics who are less equipped to assess the value and risks of cutting-edge research and care can contribute to reduce trust and avert their participation. Moreover, it can reinforce a long-lasting pattern of exclusion found across the European Region where minority and socioeconomically disadvantaged groups have been systematically under-represented in health research, as well as in the participatory spaces created to involve lay people in its design and implementation (17, 66, 67).

Assessing and attending to consent preferences and offering time and support to anyone expected to make informed decisions is essential (68, 69). However, with the exponential growth of data sources and data uses, informal support may not be sufficient to enable informed consent (44). As argued by Fiske et al. (65), it is necessary to make way for a new group of professionals—health information counselors—who can advise on the far-reaching implications of data decisions and assist in addressing arising ethical, legal and social challenges and dilemmas that often extend beyond the individual sphere (e.g., the right to choosing not to know and, thus, to decline the return of incidental research findings that may identify a predisposition for late-onset genetic diseases with implications for the offspring) (70). Health information counseling services may be especially relevant for decisions concerned with the use of one's data for purposes other than those for which it was originally collected. The reuse of health data can occur in contexts with norms and values different from those upheld in research and care settings and which are more often subject to “data trust deficits” (71). Commercial settings such as direct-to-consumer genetic testing companies are one such example where values such as transparency and reciprocity may be overridden by economic interest (e.g., patenting consumer data that was first shared under the pretense that it would be used to democratize genomics) [see (72)]. Elucidating on the ethical, legal and social implications of sharing data for research, care, commercial and other secondary purposes is of critical importance to reduce resource gaps, inform lay people's expectations, empower them to make informed decisions and promote the trustworthiness of data processing organizations.

## Strengths and Limitations

This study offers three major contributions. First, it is one of a few studies to assess public views about involvement in all key dimensions of individual-level data governance and to enable the identification of differences in the importance attributed to participation in decision-making concerned with health data sharing, access, use, and reuse. Another major contribution relates to the examination of its association with various types of trust and sociodemographic variables. Finally, data collection was carried out over an extended recruitment period of 10 months and participants were consecutively invited to participate at two reference centers for rare diseases located in an academic hospital center that oversees patients from the entire Northern Health Region of Portugal. Nevertheless, recruitment in one region limits the generalizability of the results and thus inferences for the general rare diseases population should be performed with caution. Furthermore, the value attributed to opportunities for involvement in decisions about health data sharing, access, use and reuse may be overestimated in this particular setting, as the reference centers have a strong academic orientation and are involved with rare diseases European Reference Networks. Many of the patients and carers surveyed have been involved in data sharing for national and international research projects and are experienced in decision-making concerned with their health data. However, this specific context might entail power-asymmetric relationships which may influence research participation and the data collected (73). The recruitment of participants in non-academic and in private settings would enable an enriching comparison. Finally, further qualitative and quantitative research is warranted to uncover participants' motivations and expectations regarding involvement in individual-level data governance, as well as to provide an in-depth understanding on the factors that contribute to foster and sustain public trust in research carried out in health care institutions.

## Conclusion

The high value attributed by participants to involvement in individual-level data governance stresses the need to rethink opportunities for public participation in health data decision-making. Broadening the consent options currently on offer to people affected by rare diseases to include mechanisms that allow them to choose between broad, blanket and dynamic consent models according to the type of data requested and the context in which that request is made deservers thorough consideration. Trust in science and other institutions played an important role in shaping our participants' views about involvement. Accordingly, the adoption of a meta consent approach (32) would likely need to be accompanied by the provision of transparent information about the implications of data sharing, assistance with obtaining the resources needed to make informed choices and the development of harm mitigation tools and redress.

## Data Availability Statement

The datasets generated and analyzed for this study are not publicly available due to a confidentiality agreement securing participants' privacy and anonymity, but they are available from the corresponding author upon reasonable request.

## Author Contributions

CF conceptualized the study. SS and CF designed the data analysis strategy. NN performed data analysis. NN and CF wrote the first draft of the manuscript. NN, SS, and CF contributed to the interpretation of data, critically reviewed previous versions of the manuscript, and approved the final version. All authors contributed to the article and approved the submitted version.

## Funding

This work was funded by FEDER through the Operational Programme for Competitiveness and Internationalisation and national funding from the Foundation for Science and Technology—FCT (Portuguese Ministry of Science, Technology and Higher Education) (Ref. POCI-01–0145-FEDER-032194), under the project Public and patient involvement in health data governance: a people-centred approach to data protection in genetic diseases (Ref. FCT PTDC/SOC-SOC/32194/2017) and the Unidade de Investigação em Epidemiologia—Instituto de Saúde Pública da Universidade do Porto (EPIUnit) (Ref. UIDB/04750/2020), Laboratório para a Investigação Integrativa e Translacional em Saúde Populacional (ITR) (LA/P/0064/2020), the individual contract grant DL57/2016/CP1336/CT0001 (CF) and the individual contract grant IF/01674/2015 (SS).

## Conflict of Interest

The authors declare that the research was conducted in the absence of any commercial or financial relationships that could be construed as a potential conflict of interest.

## Publisher's Note

All claims expressed in this article are solely those of the authors and do not necessarily represent those of their affiliated organizations, or those of the publisher, the editors and the reviewers. Any product that may be evaluated in this article, or claim that may be made by its manufacturer, is not guaranteed or endorsed by the publisher.
